# Examining the role of EGR1 during viral infections

**DOI:** 10.3389/fmicb.2022.1020220

**Published:** 2022-10-21

**Authors:** Caitlin M. Woodson, Kylene Kehn-Hall

**Affiliations:** ^1^Department of Biomedical Science and Pathobiology, Virginia-Maryland College of Veterinary Medicine, Virginia Polytechnic Institute and State University, Blacksburg, VA, United States; ^2^Center for Emerging, Zoonotic, and Arthropod-borne Pathogens, Virginia Polytechnic Institute and State University, Blacksburg, VA, United States

**Keywords:** early growth response protein 1, transcription, virus, herpesvirus, inflammation, Venezuelan equine encephalitis virus, alphavirus

## Abstract

Early growth response 1 (EGR1) is a multifunctional mammalian transcription factor capable of both enhancing and/or inhibiting gene expression. EGR1 can be activated by a wide array of stimuli such as exposure to growth factors, cytokines, apoptosis, and various cellular stress states including viral infections by both DNA and RNA viruses. Following induction, EGR1 functions as a convergence point for numerous specialized signaling cascades and couples short-term extracellular signals to influence transcriptional regulation of genes required to initiate the appropriate biological response. The role of EGR1 has been extensively studied in both physiological and pathological conditions of the adult nervous system where it is readily expressed in various regions of the brain and is critical for neuronal plasticity and the formation of memories. In addition to its involvement in neuropsychiatric disorders, EGR1 has also been widely examined in the field of cancer where it plays paradoxical roles as a tumor suppressor gene or oncogene. EGR1 is also associated with multiple viral infections such as Venezuelan equine encephalitis virus (VEEV), Kaposi’s sarcoma-associated herpesvirus (KSHV), herpes simplex virus 1 (HSV-1), human polyomavirus JC virus (JCV), human immunodeficiency virus (HIV), and Epstein–Barr virus (EBV). In this review, we examine EGR1 and its role(s) during viral infections. First, we provide an overview of EGR1 in terms of its structure, other family members, and a brief overview of its roles in non-viral disease states. We also review upstream regulators of EGR1 and downstream factors impacted by EGR1. Then, we extensively examine EGR1 and its roles, both direct and indirect, in regulating replication of DNA and RNA viruses.

## Introduction

Early growth response 1 (EGR1) is a transcription factor (TF) that was simultaneously identified by numerous research groups in the 1980s and as such there are several alternate names in the literature including: EGR1 (Sukhatme et al., 1988), NGFI-A (Milbrandt, 1987), Krox-24 (Lemaire et al., 1988), TIS8 (Lim et al., 1987, 1989), and Zif268 (Christy et al., 1988). The EGR1 gene itself is highly conserved between numerous species including mouse, rat, chicken, zebrafish, chimpanzee, dog, cow, and human (Havis and Duprez, 2020). EGR1 is expressed in numerous cell types and is activated transiently and rapidly by a wide array of stimuli such as growth factors, cytokines, mitogens, apoptosis, oxygen deprivation, oxidative stress, shear stress, and tissue injury (Cao et al., 1990; Ouellette et al., 1990; Hirata et al., 1998; Yan et al., 1999; Nishi et al., 2002; Pines et al., 2005; Banerji and Saroj, 2021; Khachigian, 2021). EGR1 has a broad range of physiological functions and associates with numerous cellular signaling cascades and binding partners in response to stimulation. EGR1’s primary DNA binding domain consists of three zinc finger motifs that preferentially bind to the GC-rich promoter sequence 5’-GCG(T/G)GGGCG-3′ (Christy and Nathans, 1989; Cao et al., 1990; Lemaire et al., 1990). Once induced, EGR1 is capable of transmitting activation signals downstream to influence transcriptional regulation of specific target genes required to initiate the appropriate biological response (McMahon and Monroe, 1996).

EGR1 is most notably known for its roles in the adult nervous system where it regulates critical processes underlying neuronal activity, from neurotransmission and synaptic plasticity, to higher order processes such as learning and memory, as well as reward and stress responses (Duclot and Kabbaj, 2017). EGR1 is readily expressed in various regions of the brain such as the medial prefrontal cortex, striatum, hippocampus, and amygdala and baseline expression levels of EGR1 are maintained through normal ongoing neuronal activity (Milbrandt, 1987; Herdegen et al., 1990; Mack et al., 1990; Waters et al., 1990; Aguilar et al., 2011; Duclot and Kabbaj, 2017). EGR1 is involved in the maintenance of long-term potentiation which is critical for the formation of memories, both long-term memory and for reconsolidation of memory after reactivation during retrieval (Jones et al., 2001). Altered EGR1 expression patterns have been documented in humans with neuropsychiatric disorders, like schizophrenia, where decreased EGR1 expression in the dorsal lateral prefrontal cortex is thought to contribute to patient symptoms. Furthermore, these patients also had increased EGR1 mRNA transcripts in the bloodstream (Gallo et al., 2018).

Expression of EGR1 is altered in numerous cancers where EGR1 can play paradoxical roles as either a tumor suppressor or oncogene, depending on the type of cancer and other relevant physiological circumstances. In prostate and gastric cancers, EGR1 expression is often overexpressed in the tumor as compared to surrounding tissue (Myung et al., 2013; Gabriel et al., 2016; Li et al., 2019; Ma et al., 2021). Meanwhile, EGR1 also has tumor suppressor properties through regulation of tumor suppressor genes such as transforming growth factor beta 1 (TGF1β), PTEN, and p53 (Baron et al., 2006). In gliomas and melanocytomas, EGR1 upregulates tumor suppressor gene p21^Waf1/Cip1^ which ultimately contributes to tumor cell apoptosis (Calogero et al., 2001; Schmidt et al., 2019). Low expression levels of EGR1 are also found in other tumor types, including breast, lung, and fibrosarcoma (Aliperti et al., 2019).

Numerous other disease states including cardiovascular disease, ischemia–reperfusion injury, acute lung injury, atopic dermatitis, and sepsis, among others, have been associated with EGR1 dysregulation and are reviewed elsewhere (Nishi et al., 2002; Ngiam et al., 2007; Chen et al., 2019; Khachigian, 2021; Yeo et al., 2021). Finally, EGR1 has been implicated to play a role in bacterial and viral infections; however, EGR1 and its role in viral infections have not previously been extensively reviewed. Thus, the purpose of this review is to elucidate the roles of EGR1 during viral infections.

## EGR1 structure

**Figure 1:**
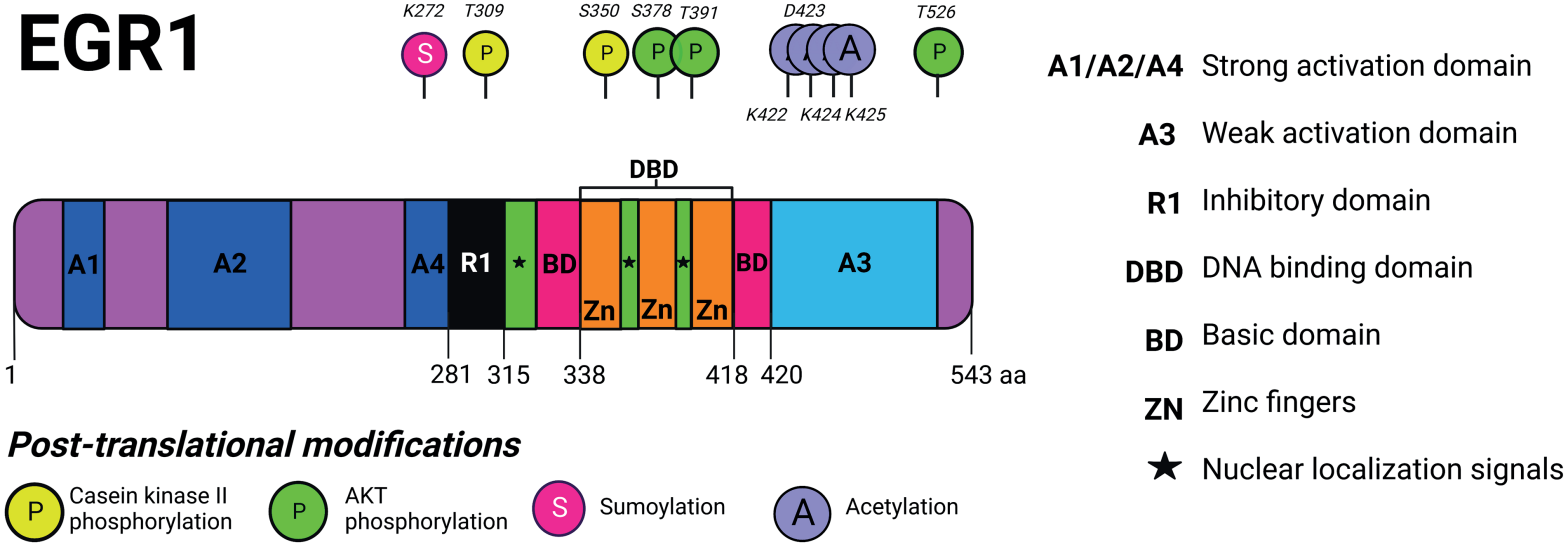
EGR1 protein domains and post-translational modifications. The multiple protein domains of EGR1 are depicted including its strong activation domain, weak activation domain, inhibitory domain, DNA binding domain, and basic domain as well as its zinc finger motifs and nuclear localization signals (NLS). EGR1 is phosphorylated by AKT on S378, T391, and T526 and by casein kinase II (CKII) on T309 and S350. EGR1 is sumoylated on K272 and acetylated on K422, D423, K424, and K425. Created with BioRender.com.

EGR1 is a zinc-finger transcription factor encoded by two exons mapped to human chromosome 5. As a modular structure, EGR1 has an extended strong activation domain on the N-terminus [amino acid (aa) 1 to 281] (Figure 1). The strong activation domain is followed by an inhibitory domain which consists of about 35 amino acids (aa281 to 315). Next is the highly conserved DNA-binding domain which is comprised of three Cys2-His2 type zinc-fingers (aa338 to 418). Within the DNA-binding domain is the nuclear localization domain which also includes bipartite nuclear localization signals (aa315 to 419). Lastly, is the weak activation domain on the C-terminus (aa420 to 543; Gashler and Sukhatme, 1995).

The inhibitory domain functions as a binding site for two transcriptional co-repressors known as NGFI-A binding proteins 1 and 2 (NAB1, NAB2) both of which are capable of suspending the biological activity of EGR1 (Russo et al., 1995; Svaren et al., 1996; Bhattacharyya et al., 2013). The prevalence of NAB1 and NAB2 in a particular cell is critical for EGR1 functionality because induction of EGR1 as a transcriptional regulator may be neutralized by NAB1 or NAB2 (Srinivasan et al., 2006). Furthermore, EGR1 directly regulates NAB2, indicating that EGR1 controls its own biological activity in a negative feedback loop *via* the synthesis of NAB2 (Havis and Duprez, 2020). Moreover, the binding of EGR1 to its own promoter leads to transcriptional repression or activation, depending on the cell type (Cao et al., 1993). For example, EGR1 regulation of itself leads to transcriptional activation in macrophages whereas in fibroblasts it leads to transcriptional repression (McMahon and Monroe, 1996). The C′ terminal weak activation domain is rich in proline, serine, and threonine residues with weak transactivation capabilities as compared to the strong N′ terminal activation domain which is rich in serine and threonine residues with strong transactivation properties (DeLigio and Zorio, 2009). Current literature lacks insight as to the structural differences between the strong and inhibitory binding domains, other than the N′ terminal being predominantly active as compared to the C′ terminal weak activation domain.

A major aspect of protein regulation in eukaryotic cells is the trafficking of proteins between the cytoplasm and the nucleus through the nuclear pore complex (NPC). EGR1 expression levels are poised at baseline in both the nucleus and the cytoplasm (albeit to a lesser extent) and increased expression in either location can be induced after exposure to various stimuli. Two nuclear localization signals (NLSs) have been identified for EGR1: one positioned at amino acids 315–330 and another between amino acids 406–417 (Chen et al., 2011). Another NLS, named SPS (a motif of three amino acids – Ser/Thr-Pro-Ser/Thr), directs target proteins to the nucleus through direct interaction with importin-7 (Imp-7) and EGR1 contains an SPS on the C′ terminus downstream of its zinc fingers. In response to growth factors, such as serum, EGR1 was found to form a complex with Imp-7 and Imp-7 was required for the nuclear translocation of EGR1. The SPS in EGR1 can serve as an independent NLS and stimulation may induce SPS to facilitate increased nuclear import of EGR1 to promote enhanced transcriptional activity in response to extracellular stimulation (Chen et al., 2011).

In terms of post-translational modifications, phosphorylation events of EGR1’s domains are controlled by protein kinases and phosphatases (Cao et al., 1992). There are at least five known phosphorylation sites that are regulated by casein kinase II phosphorylation (S378, T391, T526) or AKT phosphorylation (T309, S350) as depicted in Figure 1. Phosphorylation of the different EGR1 domains can either enhance or suspend the transcriptional activity of EGR1. Following UV irradiation in a fibrosarcoma model, EGR1 is phosphorylated by both protein kinase C (PKC) and tyrosine kinases and protects cells from apoptosis (Huang et al., 1998). Meanwhile, phosphorylation by casein kinase II in fibrosarcoma cells decreases the transcriptional activity of EGR1 as well as its DNA binding activity (Jain et al., 1996). In terms of acetylation, the cyclic AMP responsive element binding protein (CREB)-binding protein (CBP)/p300 complex can acetylate EGR1 within its weak activation domain (K422, D423, K424, K425; Yu et al., 2004). EGR1 can also undergo sumoylation (K272) and ubiquitination following translation which has been reviewed elsewhere (Havis and Duprez, 2020). EGR1’s functionality as an activator or repressor of target genes is dependent on its post-translational modifications which modulate its activity.

## Early growth response family members

EGR1 belongs to the EGR family of C2H2-type zinc finger proteins which also include EGR2, EGR3, and EGR4 (Beckmann and Wilce, 1997). EGR family members are rapidly induced in response to environmental and cellular stimuli and are also transcriptional regulators capable of activating specialized signaling cascades. All four EGR family members contain three cystine2-histidine2 zinc fingers and are highly homologous both within and between species suggesting possible target and functional overlap (Duclot and Kabbaj, 2017). Furthermore, alignment of the DNA binding domains of the EGR family members from humans, rats, and mice suggests that differences between family members are greater within species than between species which suggests that the characteristics of each EGR member are evolutionarily conserved (Duclot and Kabbaj, 2017). However, with the exception of their shared DNA-binding domain, the sequences between the family members are much less homologous suggesting specificities in protein–protein interactions allowing for differences in gene regulation, reactivity, and transcriptional control (Poirier et al., 2008). EGR2 is most notably associated with the onset of myelination within the peripheral nervous system as well as hindbrain segmentation (Warner et al., 1998; Taefehshokr et al., 2017). Defects in EGR2 expression can clinically manifest as various forms of neuropathies such as Charcot–Marie–Tooth disease. Meanwhile, EGR3 is associated with muscle-spindle development, lymphocyte development, endothelial cell growth and migration, and neuronal development. EGR3 dysfunction has been implicated in psychiatric disorders such as schizophrenia and bipolar disorder (Pfaffenseller et al., 2018). EGR4 is a paralog of EGR2 and has been implicated in posterior hindbrain development and also plays a critical role in spermatogenesis in male murine fertility (Bae et al., 2015; Taefehshokr et al., 2017). In alignment with the other EGR family members, EGR4 defects are associated with psychiatric disorders such as schizophrenia as well as neuropathies. Outside of the nervous system, EGR1, EGR2, and EGR3 have been implicated to be critical for response to external stimuli and to direct lineage differentiation within the immune system (Sun et al., 2019).

## Upstream regulators of EGR1

**Figure 2:**
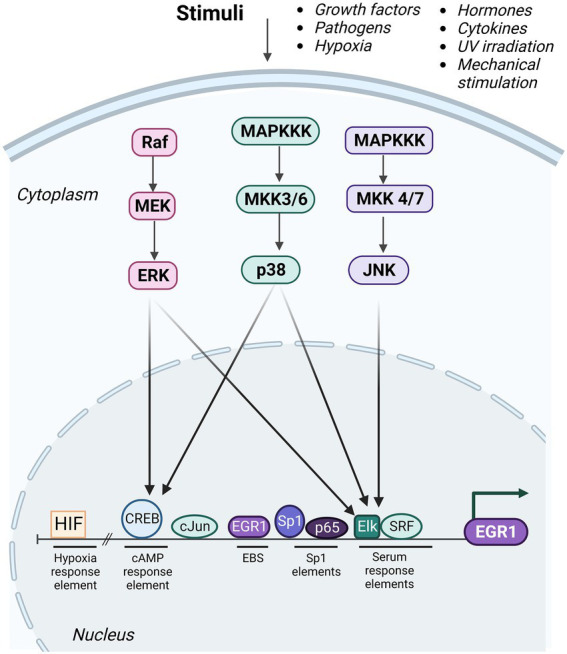
Upstream regulators of EGR1. The various regulatory elements of the EGR1 promoter are depicted including the hypoxic response element (HIF), cyclic adenosine 3′, 5′-monophosphate (cAMP) response elements (CRE), EGR1 binding sites (EBS), specificity protein 1 (Sp1) elements, and serum response elements (SREs). Multiple transcription factors are capable of binding to these elements to regulated EGR1 expression including HIF, cAMP response element binding protein (CREB), cJun, EGR1, Sp1, nuclear factor kappa B (NF-κB)/p65, Elk, and serum response factor (SRF). Multiple stimuli induce activation of ERK, JNK, and p38 MAPK signaling cascades, which results in activation of Elk and/or CREB transcription factors and induction of EGR1 gene expression. Created with BioRender.com.

The promoter region of the EGR1 gene contains transcription factor binding sites such as cyclic adenosine 3′, 5′-monophosphate (cAMP) response elements (CRE), an activator protein-1 (AP-1) binding site, an EGR binding site (EBS), specificity protein 1 (Sp1) elements, nuclear factor kappa B (NF-κB) binding site, and serum response elements (SREs; Figure 2). The CREs region can be occupied by members of the CREB protein family of transcription factors (Banerji and Saroj, 2021). The SREs function as binding sites for serum response factors (SRFs) and ternary complex factors (TCFs), such as Elk-1. As a bivalent regulator of EGR1, Elk-1 can induce transcription of EGR1 through recruitment of histone acetyltransferases, such as p300/CBP (Li et al., 2003), or function to repress EGR1 transcription *via* recruitment of histone deacetylases, such as mSin3A-HDAC (Yang et al., 2001). The EGR1 promoter also contains two CpG islands that are susceptible to DNA methylation. Promoters with methylated CpG regions generally correspond with repressed gene expression. However, due to its structural plasticity EGR1 is capable of binding to its target DNA sequence regardless of methylation state and is capable of binding to target regions that are completely methylated by adapting its conformational structure to not only recognize the target DNA but also maintain its affinity for the target sequence (Zandarashvili et al., 2015).

Activation of EGR1 gene expression is induced by numerous upstream signal transduction pathways that are activated *via* external stimuli and subsequently these pathways direct molecules to the EGR1 promoter (de Klerk et al., 2017; Figure 2). Mitogen-activated protein kinases (MAPKs), protein kinase A (PKA), protein kinase B (PKB)/AKT, PKC, and NF-κB signaling factors have all been shown to regulate EGR1 gene expression (Havis and Duprez, 2020; Banerji and Saroj, 2021; Khachigian, 2021). The most well-characterized signal transduction cascade that impacts EGR1 transcriptional regulation is the MAPK pathway. MAPKs are ubiquitously expressed phosphorylated protein kinases that are known to regulate a variety of cellular activities such as stress response, apoptosis, and genes required for immune response induction. The MAPK pathway is part of an extensively integrated signaling network and MAPK signaling regulates many physiological responses that vary according to stimulus and cell type (Johnson, 2011). All MAPK pathways are composed of three core signaling modules including the Extracellular Signal-Related Kinase (ERK1/2), the c-Jun NH2-terminal kinase (JNK), and p38 MAPK (Kyriakis and Avruch, 2001). ERK1/2 is activated in response to growth factors, hormones, and various pro-inflammatory stimuli while cellular and environmental stressors activate JNK1/2/3 and p38 MAPK pathways (Banerji and Saroj, 2021). Previous studies have shown that EGR1 can be transcriptionally regulated *via* ERK-dependent phosphorylation of Elk-1, which as mentioned above can result in either target gene activation or repression, depending on the epigenetic modifications of the EGR1 promoter. Genome-wide microarray analysis revealed EGR1 as the most upregulated gene following JNK activation and c-Jun as an essential effector in EGR1 transcriptional regulation (Hoffmann et al., 2008). c-Jun directly binds the AP-1 element and is required for EGR1 promoter activation through the three distal SREs (Hoffmann et al., 2008). Activated p38 can directly induce transcription factors such as CREB to bind to its respective binding site on the EGR1 promoter and thus induce EGR1 upregulation (Bachstetter and Van Eldik, 2010).

Transcriptional regulation of EGR1 is a complex and dynamic process especially considering its many interactions between transcription factor binding, cofactor recruitment, and chromatin modifications including histone methylation, acetylation, phosphorylation, and nucleosome remodeling (Duclot and Kabbaj, 2017). Exposure to phorbol esters rapidly induces EGR1 expression within minutes but returns to baseline transcriptional levels by 180 min after exposure. Riffo-Campos et al. propose a model in which Elk1, CREB and EGR1 co-localize to rapidly induce EGR1 transcription (Riffo-Campos et al., 2015). Three components of histone deacetylase (HDAC) complexes are found on the EGR1 promoter prior to exposure to phorbol esters. However, CREB, Elk-1, SRF and RNA polymerase II can also be found at the EGR1 promoter prior to its induction thus corroborating the understanding that EGR1 is poised at baseline. This can be partially attributed to favorable nucleosome positioning. Phorbol ester exposure triggers classical nucleosome repositioning and triggers partial eviction of the +1 and − 1 nucleosomes as well as sliding of the – 2 nucleosome at 15 min post-exposure. Simultaneously, CREB and Elk1, both bound to the EGR1 promoter, are phosphorylated in a p38- and MEK1/2- dependent manner (Tur et al., 2010) which triggers an increase in phosphorylation (pS10) and acetylation (AcK14) of histone H3 at the +1 nucleosome (Riffo-Campos et al., 2015) and is likely mediated by the transcriptional co-factor CBP. Altogether, EGR1 gene regulation is dependent on a variety of factors such as binding partners bound to its promoter and upstream signaling pathways, including the MAPK cascades (Banerji and Saroj, 2021; Figure 2).

## Downstream targets of EGR1

There are many potential downstream targets of EGR1 and it was initially assumed that genes containing EGR1 binding sites were thought to be directly regulated by EGR1 (Swirnoff and Milbrandt, 1995). However, experimental evidence found that EGR1 can also regulate gene expression indirectly through interacting with other transcription factors such as c/EBPβ, Fos, or Jun, thereby further expanding the range of potential EGR1 targets and biological processes regulated by EGR1 (Duclot and Kabbaj, 2017). Initially, identification of EGR1 dependent genes was investigated on a single-gene basis. The development of genomic wide screening technologies enabled the identification of EGR1 regulated genes on a global scale. However, it is important to keep in mind that many of the genes identified to be regulated by EGR1 are likely specific to the cell type and insult encountered prior to samples undergoing sequencing. Results gained from the Encyclopedia of DNA Elements (ENCODE) project identified approximately 8,552 of 15,872 genes annotated (~54%) contain at least one EGR1 binding region within 3 kb of their transcriptional start sites (Consortium EP, 2012). These studies further corroborated previous speculation that EGR1 can bind a large number of genes across several human cell types and thus exert transcriptional regulation for a wide variety of biological functions. Functional analysis of genes identified in the ENCODE project revealed enrichment of pathways and processes related to growth factor signaling and intracellular signaling cascades. The molecular functions of EGR1-bound genes were found to range from chromatin and transcription factor activity to guanyl-nucleotide exchange factor activity through serine/threonine kinase activity. Moreover, EGR1-bound target genes were localized from the chromatin to the cell membrane (Duclot and Kabbaj, 2017). Similarly, EGR1 ChIP-seq studies of mouse brains identified enrichment of biological processes and pathways related to protein trafficking, synaptic vesicle transport, endocytosis, protein phosphorylation, and intracellular signaling cascades (Koldamova et al., 2014).

**Figure 3:**
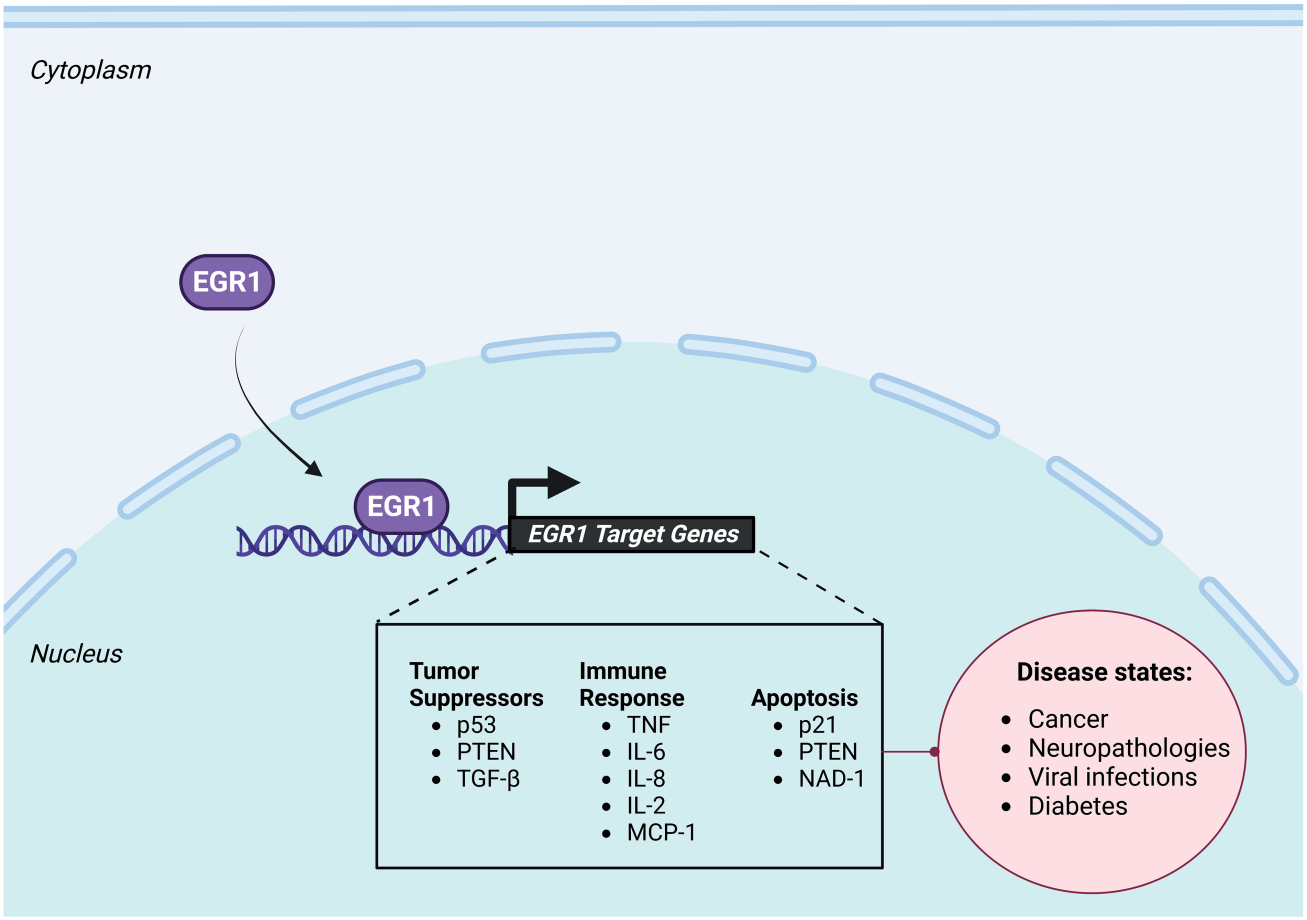
Downstream Targets of EGR1. EGR1 induces transcription of a large repertoire of genes, including tumor suppressors, immune response genes, and apoptotic factors. Changes in EGR1 dependent gene expression have been correlated with multiple disease states including cancer, diabetes, neuropathologies, and viral infections. Created with BioRender.com.

Numerous tumor suppressor genes have been identified to be under the transcriptional regulation of EGR1 (Figure 3). TGFβ1, a protein critically involved in regulation of cell proliferation, differentiation, and growth, is directly regulated by EGR1 in human fibrosarcoma cells (Liu et al., 1996; De Belle et al., 2000) and monkey kidney cells (Dey et al., 1994). Furthermore, fibronectin, a downstream target of TGFβ1/Smad signaling pathway, was also found to be a direct target of EGR1, with two closely spaced EBS located within its proximal promoter (Liu et al., 2000). Phosphatase and tensin homolog (PTEN), a proapoptotic factor that is altered in numerous cancers, is also directly regulated by EGR1. *In vivo* studies of wild-type mice exposed to irradiation indicated a strong upregulation of EGR1 and PTEN mRNA. However, EGR1 knock-out mice exposed to irradiation did not result in an upregulation of PTEN, indicating that EGR1 directly regulates PTEN *in vivo* (Virolle et al., 2001). Additionally, an extensive review of clinical cases of human non-small-cell lung cancer found that EGR1 expression levels are predictive of both PTEN expression levels and survival outcome with a high degree of significance (Baron et al., 2006). EGR1 has also been identified as a regulator of p53 in both humans and mice, both of which contain EBS within their promoter regions. Loss of EGR1 in mouse embryonic fibroblasts (MEFs) suppresses p53-dependent growth regulatory functions (Krones-Herzig et al., 2003) and ChIP and EMSA assays confirmed EGR1 binding to the p53 promoter both *in vitro* and *in vivo* (Krones-Herzig et al., 2005).

EGR1 is known to target immunologically significant genes, such as tumor necrosis factor (TNF; Figure 3). As a cytokine, TNF plays an important role in the early phases of the immune response by promoting inflammation. As such, the promoter region of TNF is GC-rich and contains a binding site for EGR1 (McMahon and Monroe, 1996). Additionally, previous studies in RAW 264.7 cells showed that following LPS-induced ERK1/2 activation, TNFα mRNA is upregulated in an EGR1 dependent manner and that EGR1 binds to the TNFα promoter (Shi et al., 2002). Moreover, the promoter of the inflammatory gene IL-2 was found to have an overlapping Sp1 and EBS and EGR1, in combination with NFAT, was found to upregulate the expression of IL-2 following LPS stimulation (Decker et al., 1998). *In vivo* ChIP analysis revealed EGR1 regulates additional proinflammatory mediators such as IL-8, IL-6, and monocyte chemoattractant protein 1 (CCL2/MCP-1; Hoffmann et al., 2008).

EGR1 has also been implicated in apoptosis (Figure 3), most notably in cancer studies where EGR1 expression is significantly reduced in developing tumors (Huang et al., 1997). Upon exposure to damaging ionizing radiation EGR1 binds to the 5’ UTR of the PTEN gene, which contains an EBS, a tumor suppressor and pro-apoptotic gene, to induce apoptosis (Virolle et al., 2001). Additionally, EGR1 was shown to activate transcription of p21^Waf1/Cip1^, a gene involved in DNA damage response, differentiation, senescence, and apoptosis, independently of p53 in response to curcumin treatment in human astrocytoma cells (Choi et al., 2008). Another pro-apoptotic gene, NSAID-activated gene 1 (NAD-1), is regulated by EGR1 and has been shown to facilitate apoptosis in colon carcinoma cells, lung cancer cells, and hepatocellular carcinoma cells (Chen et al., 2007; Shin et al., 2008).

## EGR1 and viral infections

Host-pathogen interactions are dynamic and complex. Throughout their co-evolution, hosts have acquired ways to overcome viral infection and likewise viruses have developed countermeasures to overcome the host’s antiviral cellular state. Viruses, with their relatively compact genomes and high replication rates, are prone to mutations and thus continuously present the host with ever-evolving challenges. To combat the host antiviral response, viruses encode mechanisms to control interferon signaling as well as disrupt cross-talk between cellular pathways that are known to induce apoptosis and inflammation. By utilizing numerous evasion strategies viruses are also able to subvert host cellular processes such as cell cycle regulation, major histocompatibility complex restricted antigen presentation, intracellular protein transport, apoptosis, interferon signaling, and humoral immune responses (Fan et al., 2018). A primary evasion strategy by viruses is direct interaction of viral proteins with host proteins to inhibit their functionality and by doing this the virus can hijack the cells biosynthetic machinery in order to enhance viral replication in a variety of ways such as inducing posttranslational modifications and interfering with host restriction factors to regulate their function and/or degradation. Emergence of functional genomic, proteomic, and bioinformatic technologies has allowed for more-thorough studies to aid in the quest of identifying host factors critical for viral replication and potential novel targets that can be exploited for therapeutic development. EGR1 has been associated with multiple viral infections such as VEEV (Venezuelan equine encephalitis virus), KSHV (Kaposi’s sarcoma-associated herpesvirus), HSV-1 (herpes simplex 1), JCV (human polyomavirus JC virus), HIV (human immunodeficiency virus) and EBV (Epstein–Barr virus). During viral infections, EGR1 expression can facilitate either an anti-viral or pro-viral cellular state, depending on the virus and the transcriptional events regulated by EGR1. This information is summarized in Tables 1, 2, including the cell types where the findings were discovered.

**Table 1:** DNA viral infections implicating EGR1.

Virus (Acronym)	Viral Family	Impact on EGR1	Proposed functionality	Cell type	References
JC virus (JCV)	*Polyomaviridae*	Activation	Binds to viral promoter to enhance late viral gene expression	U87MG	Romagnoli et al. (2008)
Herpes simplex virus type-1 (HSV-1)	*Herpesviridae*	Activation	Enhances viral gene expression, replication, and release of infectious virions	SIRC, Vero, HEK293T	Bedadala et al. (2007, 2011), Hsia et al. (2013)
Bovine herpesvirus 1 (BHV-1)	*Herpesviridae*	Activation	Enhances viral replication	MDBK, HEK-293 T	Hou et al. (2019)
Kaposi’s sarcoma associated herpesvirus (KSHV)	*Herpesviridae*	Activation	Enhances virion production by binding to promoter of replication and transcription activator (RTA) and suppressing RTA transcription	BC-3, BCBL-1, HEK-293 T, THP-1, HUVEC	Sarkar and Verma (2017)
Epstein–Barr virus (EBV)	*Herpesviridae*	Activation	Suppresses lytic replication	Germinal Center B cells, Burkitt’s lymphoma cells	Vockerodt et al. (2013), Chang et al. (2006), Kim et al. (2007)
Vaccinia Virus (VCV)	*Poxviridae*	Activation	Enhances infectivity	MEFs	de Oliveira et al. (2018)
Cytomegalovirus (CMV)	*Herpesviridae*	Activation	Binds to UL138 to establish latency	Fibroblasts, CD34+ HPCs, HEK-293 T	Buehler et al. (2019)

**Table 2:** RNA viral infections implicating EGR1.

Virus (Acronym)	Viral family	Impact on EGR1	Proposed functionality	Cell type	References
Enterovirus D68 (EVD68)	*Picornaviridae*	Activation	Unknown	RD	Si et al. (2021)
Seneca Valley virus (SVV)	*Picornaviridae*	Repression	Enhances interferon signaling and decreases viral replication	PK-15, HEK293T	Zhu et al. (2018)
Enterovirus 71 (EV71)	*Picornaviridae*	Activation	EGR1 binds to EV71 5’UTR to facilitate IRES activity, viral replication, and protein expression	RD, HEK-293 T, SK-N-SH	Song et al. (2015)
Foot-and-mouth disease virus (FMDV)	*Picornaviridae*	Repression	Enhancing interferon signaling *via* TBK1 phosphorylation resulting in decreased viral replication	PK-15, HEK293T	Zhu et al. (2018)
Borna disease virus (BDV)	*Bornaviridae*	Activation	Unknown	Rat brain (*ex vivo*)	Fu et al. (1993)
Human T-cell leukemia virus type 1 (HTLV-1)	*Retroviridae*	Activation	Enhances NF-kB signaling induced by HTVL-1 Tax	Jurkat, Hela, TaxP, TaxN, HEK-293 T, JPX-9	Fujii et al. (1991), Huang et al. (2017)
Human immunodeficiency virus-1 (HIV)	*Retroviridae*	Activation	Drives Tat-dependent HIV transcription in latency reversal	CD4+ T cells, Jurkat, J-Lat A1, TZM-bl, CEM	Dron et al. (1999), Wong et al. (2022)
Lymphocytic choriomeningitis virus (LCMV)	*Arenaviridae*	Repression	Unknown	Macaque PBMC (*ex vivo*)	Djavani et al. (2007)
Human foamy virus (HFV)	*Retroviridae*	Activation	Unknown	HEL299	Wagner et al. (2000)
Venezuelan equine encephalitis virus (VEEV)	*Togaviridae*	Activation	Enhances virally induced cell death through regulation of inflammatory mediators and apoptotic machinery	U87MG, MEFs	Baer et al. (2016), Dahal et al. (2020). Lehman et al. (2022)
Chikungunya virus (CHIKV)	*Togaviridae*	Activation	Unknown	U87MG	Lehman et al. (2022)
Eastern equine encephalitis virus (EEEV)	*Togaviridae*	Activation	Unknown	U87MG	Lehman et al. (2022)
Sindbis virus (SINV)	*Togaviridae*	Activation	Unknown	U87MG	Lehman et al. (2022)
Japanese encephalitis virus (JEV)	*Flaviviridae*	Activation	Unknown	Mouse brain (*ex vivo*)	Saha and Rangarajan (2003)
Zika virus (ZIKV)	*Flaviviridae*	Activation	Unknown	U87MG	Lehman et al. (2022)
Rift Valley fever virus (RVFV)	*Phenuiviridae*	Activation	Unknown	U87MG	Lehman et al. (2022)
Rabies virus	*Rhabdoviridae*	Activation	Unknown	Rat brain (*ex vivo*)	Lehman et al. (2022)
Avian influenza (H5N1)	*Orthomyxoviridae*	Repression	Unknown	NHBE	Tatebe et al. (2010)
Infectious bronchitis virus (IBV)	*Coronaviridae*	Activation	Suppresses host antiviral response, survival factor in virally induced apoptosis	H1299, Vero	Yuan et al. (2022)
Porcine epidemic diarrhoea virus (PEDV)	*Coronaviridae*	Activation	Suppresses host antiviral response, survival factor in virally induced apoptosis	H1299, Vero	Yuan et al. (2022)
Human coronavirus-OC43 (HCoV-OC43)	*Coronaviridae*	Activation	Suppresses host antiviral response	H1299, Vero	Yuan et al. (2022)
Murine coronavirus mouse hepatitis virus (MHV)	*Coronaviridae*	Activation	Represses pro-apoptotic gene BNip3	DBT	Cai et al. (2006)
Human coronavirus-229E (HCoV-229E)	*Coronaviridae*	Activation	Inhibits viral replication	H1229	Yuan et al. (2022)
Porcine epidemic diarrhoea virus (PEDV)	*Coronaviridae*	Activation	Suppresses PEDV replication through degradation of the viral N protein	LLC-PK1, Vero, HEK-293 T	Wang et al. (2021)
Severe acute respiratory syndrome coronavirus 2 (SARS-CoV-2)	*Coronaviridae*	Activation	Unknown	Clinical samples	Domdom et al. (2020)
Fowl adenovirus serotype 4 (FAdV-4)	*Adenoviridae*	Activation	Unknown	LMH	Wang et al. (2022)

### DNA viruses

#### Polyomaviridae

JC virus (JCV), also known as human polyomavirus 2, is the causative agent of the fatal demyelinating disease known as progressive multifocal leukoencephalopathy (PML). EGR1 is upregulated in human astrocytoma cells infected with JCV and binds to the JCV late promoter to enhance viral transcription (Romagnoli et al., 2008). Moreover, mutation of the EBS impaired the ability of EGR1 to bind to the viral promoter and resulted in significant reduction in late gene expression and DNA replication during the course of infection (Romagnoli et al., 2008). Importantly, EGR1 was found to be upregulated in PML clinical samples and immunohistochemistry staining revealed the strong presence of EGR1 in the nuclei of oligodendrocytes, the primary site of JCV infection and replication (Romagnoli et al., 2008). These results suggest that EGR1 induction following infection with JCV may play an important role in JCV replication and PML pathogenesis.

#### Herpesviridae

EGR1 upregulation has been documented in several herpesviruses including HSV-1, bovine herpesvirus 1 (BHV-1), KSHV, EBV, and human cytomegalovirus (CMV). HSV-1 is a neurotropic virus and initial infection, which is usually mild or asymptomatic in nature, typically occurs during childhood. HSV-1 infections of epithelial cells initiates lytic replication upon which virions infect neurons in the trigeminal ganglia ultimately resulting in lifelong latency (Taylor et al., 2002). An EBS was previously found in the intron of HSV-1 viral gene ICP22 and EGR1 was found to regulate viral genes ICP4 and ICP22 (Bedadala et al., 2007). More recently, EGR1 has been shown to be upregulated at both the mRNA and protein level following infection with HSV-1 in Vero cells (Bedadala et al., 2011). Importantly, EGR1 was shown to enhance viral gene expression, viral replication, and release of infectious particles. Mechanistic studies involving ChIP assays identified NF-κB and phosphorylated CREB (pCREB) as being bound to the EGR1 promoter following HSV-1 infection in rabbit corneal (SIRC) cells, but not HEK-293 T cells, thus suggesting NFκB and pCREB may play a role in regulating EGR1 upregulation during HSV-1 infections (Bedadala et al., 2011). Clinical infections of HSV-1 can progress to herpes encephalitis and induce permanent neurological damage. Given the many roles EGR1 plays within the brain in terms of neuronal plasticity as well as memory and learning, it would be interesting to further examine the role of EGR1 in the development of HSV-1 induced neurological sequalae.

BHV-1 is considered an economically important pathogen to the cattle industry worldwide. Clinical infections of BHV-1 manifest in a variety of pathologies including respiratory symptoms, gastrointestinal symptoms, genital disorders, and abortions. RNA sequencing of BHV-1 infected MDBK cells identified EGR1 as being differentially expressed and upregulated following infection and EGR1 induction was confirmed at both the mRNA and protein level (Hou et al., 2019). Importantly, EGR1 was found to enhance viral replication in BHV-1 infected MDBK cells, thus suggesting EGR1 plays a pro-viral role during BHV-1 infections. Additionally, EGR1 was found to bind to an EBS within the promoter region of the viral gene UL46 (Hou et al., 2019), but not ICP22, which differs from previous findings that EGR1 binds and regulates ICP22 in HSV-1, however this is not entirely unexpected considering they are different viruses with distinct differences in tropism and biology (Bedadala et al., 2011). Moreover, EGR1 was found to be a direct target of cellular micro-RNA bta-miR-2,361, which was also found to inhibit viral replication through downregulation of EGR1 (Hou et al., 2019). These results suggest a potential therapeutic role for bta-miR-2,361 in mitigating BHV-1 infections by directly targeting EGR1.

EGR1 has also been found to regulate the replication and transcription activator (RTA) gene, which regulates the switch from latent to lytic replication during KSHV infection (Dyson et al., 2010, 2012). Loss of EGR1 *via* small interfering RNA (siRNA) resulted in decreased virion production following lytic reactivation (Sarkar and Verma, 2017) suggesting a pro-viral role for EGR1 during KSHV infections. Previous studies suggested a role for CBP/p300 in modulating the RTA promoter activity (Gwack et al., 2001) and since EGR1 has a specific binding site for CBP/p300 it was speculated that EGR1 and CBP/p300 could be working cooperatively to regulate RTA activity. EGR1 was not essential to activate the RTA promoter however, expression of EGR1 significantly enhanced activation of the RTA promoter thus suggesting that EGR1 can regulate the transcriptional activities of CBP/p300 (Sarkar and Verma, 2017). These results suggest a potential role for EGR1 as a key regulator for KSHV lytic reactivation. In line with these results, there are also studies suggesting that EGR1 plays a role in regulating Epstein–Barr virus (EBV) lytic cycle activation (Chang et al., 2006; Vockerodt et al., 2013). EBV latent membrane protein 2A (LMP2A) maintains EBV latency (Miller et al., 1994, 1995) and EGR1 is significantly upregulated in primary human germinal center B cells transfected with LMP2A (Fujii et al., 1991). However, EGR1 upregulation was not observed following LMP2A expression in Hodgkin’s lymphoma cells lacking a functional B cell receptor, which is correlated with a lack of viral lytic replication in these cells (Vockerodt et al., 2013). Expression of EBV latent membrane protein 1 (LMP1), which is required for EBV B cell immortalization (Young and Rickinson, 2004), also induces EGR1 upregulation in a NF-κB dependent manner (Kim et al., 2007). EGR1 is upregulated in tumor samples from patients with extranodal natural killer T-cell lymphoma (ENKTL), which is an aggressive cancer associated with EBV infection (Lee et al., 2021). EGR1 expression was found to have prognostic value, with EGR1 being upregulated in the ENKTL low risk group. Moreover, EGR1 has been shown to regulate UL138, the latency gene, in CMV infections. CMV interferes with host cell signaling and downregulates epidermal growth factor receptor (EGFR) and its downstream pathways during early stages of infection in order to establish latency (Kim et al., 2017). During CMV infection, EGR1 is induced downstream of EGFR signaling *via* the MEK/ERK pathway (Buehler et al., 2016). EGR1 binds to the CMV latency gene, UL138, which stimulates its expression and consequently reinforces a latent infection state. CMV mutant viruses lacking EGR1 binding sites fail to establish latency. Thus, antagonizing the EGFR/EGR1 signaling enables CMV to maintain a productive infection (Buehler et al., 2019) and suggests EGR1 serves a pro-viral role during CMV infections.

#### Poxviridae

Infection with vaccinia virus (VACV), a member of the *Poxviridae* family, induces MAPK pathway activation which subsequently activates EGR1. Activation of MEK/ERK/EGR1 pathway enhances VACV replication in certain cell lines, including fibroblasts. Loss of EGR1 *via* siRNA targeting EGR1 or knock-out cells results in a ~ 1 log_10_ reduction of VACV replication in serum starved cells (de Oliveira et al., 2018). Furthermore, virions derived from infected EGR1 null MEFs were shown to have reduced infectivity as compared to virions derived from infected wild-type MEFs. Taken together, these results suggest a pro-viral role for EGR1 during VACV infection.

### RNA viruses

#### Picornaviridae

Enterovirus 71 (EV71) infections in young children can manifest clinically from asymptomatic, to mild hand, foot and mouth disease, to severe neurological disease. EGR1 is upregulated following infection with EV71 in RD and SK-N-SH cells and was found to facilitate viral replication (Song et al., 2015). Moreover, EGR1 directly interacts with the 5’UTR of EV71 in the cytoplasm of infected cells to facilitate viral replication in a miR-141-independent manner. Previous studies suggested that EGR1 facilitates EV71 virus production through activation of miR-141 and suppression of eIF4E (Ho et al., 2011). However, Song et al. identified an additional mechanism in that EGR1 binds to both the cloverleaf and step-loop IV secondary structures of the EV71 IRES, which contribute to viral replication and viral protein translation, respectively (Song et al., 2015). Thus, EGR1 seemingly plays a multi-functional pro-viral role in EV71 infections.

Conversely, EGR1 appears to have an antiviral effect during other picornavirus infections. EGR1 was found to be activated irrespective of IFN-β treatment following infection with foot and mouth disease virus (FMDV) in PK-15 and HEK-293 T cells (Zhu et al., 2018). Knockdown of EGR1 *via* siRNA considerably enhanced expression of the viral protein VP1, a protein involved in humoral immune response and the regulation of interferon production (Zhu et al., 2018; Peng et al., 2020). Moreover, loss of EGR1 resulted in increased viral mRNA which correlated with an increase in viral titers, thus suggesting an antiviral role for EGR1 during FMDV infection (Zhu et al., 2018). Mechanistic studies determined that EGR1 enhanced type I IFN signaling and suppressed viral replication. Moreover, EGR1 enhanced type I IFN signaling and suppressed viral replication in Seneca Valley virus (SVV), another picornavirus, which further corroborates an antiviral role for EGR1 for these viruses under these conditions (Zhu et al., 2018).

#### Retroviridae

In the early 1990’s, EGR1 was found to be upregulated in human T-cell leukemia virus type 1 (HTLV-1) and HTLV-2 infected T cells (Mallett et al., 2012). Further, the viral protein Tax was found to modulate cellular gene expression *via* CREB/activating transcription factors (ATF)-, SRF- and NF-κB-associated pathways (Matsuoka and Jeang, 2007). More recent studies have shown that HTLV-1 infection, and Tax more specifically, upregulates EGR1 expression in infected T-cells (Huang et al., 2017). Moreover, using a luciferase reporter plasmid system, it was shown that both the SRE element and NF-κB binding sites within the EGR1 promoter are essential for regulating EGR1 transcription and Tax directly binds to the NF-κB binding site within the EGR1 promoter (Huang et al., 2017). Consequently, HTLV-1 infection and dysregulation of EGR1 in infected cells was found to be regulated by Tax-induced persistent activation of the NF-κB pathway (Huang et al., 2017). Following Tax binding to the NF-κB binding site within the EGR1 promoter, upregulation of EGR1 results in NF-κB activation, which enhances EGR1 transcription *via* a positive feedback loop. The establishment of this positive feedback loop further enhances Tax-induced constitutive NF-κB activation in HTLV-1 infected cells, promoting T cell transformation and ultimately adult T-cell leukemia/lymphoma pathogenesis (Huang et al., 2017).

EGR1 is also upregulated in human immunodeficiency virus-1 (HIV-1) infected U937 monocytic cells (Dron et al., 1999). A common therapeutic strategy in treating HIV-1 infections is to “kick and kill” whereby latency reversal agonists, including protein kinase C agonists (PKCa), are employed to reactivate latent HIV-1 reservoirs so that they can be cleared by viral cytopathic effect or immune-mediated clearance. Using a Jurkat T-cell model of HIV latency, EGR1 was found to be robustly upregulated following treatment with various PKCa and a linear regression analysis of EGR1 induction compared to HIV reactivation revealed that EGR1 is highly correlated with PKCa induced HIV-1 reactivation (Wong et al., 2022). Moreover, EGR1 was shown to directly interact with the HIV-1 promoter to induce HIV-1 transcription and/or latency reversal, however further investigation is required to tease out the underlying molecular mechanisms (Wong et al., 2022). Altogether, these studies suggest a pro-viral role for EGR1 in retroviral infections which makes EGR1 an attractive target to exploit in terms of therapeutic development.

#### Coronaviridae

EGR1 appears to play paradoxical roles when it comes to coronavirus infections. There are numerous studies involving coronaviruses where activated EGR1 seemingly plays a pro-viral role. Mouse hepatitis virus (MHV) is a coronavirus capable of inducing significant damage to the CNS and is often used as an animal model for modeling multiple sclerosis in humans due to the similarities in demyelination pathology (Lampert et al., 1973; Herndon et al., 1977). EGR1 is upregulated following MHV infection of DBT cells which are mouse primary astrocyte cells (Cai et al., 2006). EGR1 was found to be activated *via* ERK1/2 signaling and loss of EGR1 *via* siRNA resulted in a significant decrease in viral replication (~2 log_10_) suggesting EGR1 may play a role in viral replication (Cai et al., 2006). More recent studies found EGR1 mRNA and protein levels are upregulated following infection with infectious bronchitis virus (IBV) in H1299, Vero, and DF1 cells (Yuan et al., 2022). Other coronaviruses examined, including porcine epidemic diarrhea virus (PEDV), human coronavirus (HCoV)-229E, and HCoV-OC43, were also found to activate EGR1 at both the mRNA and protein level following infection in both H1299 and Vero cells (Yuan et al., 2022). Following siRNA-mediated knock-down of EGR1, it was shown that loss of EGR1 has no significant impact on IBV replication but knock-down cells had significantly higher percentages of PARP cleavage, an indicator of cell death, which suggests that EGR1 may function as a survival factor during IBV infection of H1299 cells (Yuan et al., 2022). Knock-down of EGR1 resulted in slightly, but not significantly, reduced replication of PEDV and increased PARP cleavage, similar to results observed with IBV. Moreover, the effects of EGR1-knockdown on the expression of cFos and cJun, both AP-1 family members, was also examined and loss of EGR1 resulted in drastically reduced induction of both cFos and cJun, albeit more so at later timepoints (Yuan et al., 2022). The observation that IBV infection induces a mutual regulatory effect on both EGR1 and AP-1 genes propelled the authors to determine the upstream kinases that potentially regulate EGR1 expression (Yuan et al., 2022). To this end, small molecule inhibitors targeting ERK1/2, JNK, or p38 pathways were utilized and results indicated that ERK1/2 may function as an upstream regulator of EGR1 during IBV, PEDV, and HCoV-229E coronavirus infections (Yuan et al., 2022). Overall, these results suggest that EGR1 may serve a pro-viral function during coronavirus infections through suppression of the host antiviral response and potentially as a survival factor in virally induced apoptosis in PEDV and IBV infections.

EGR1 has been implicated in multiple pandemic viruses including SARS-CoV and SARS-CoV-2. Studies have also shown that EGR1 upregulates TGF-β1 *via* activation of ROS/p38 MAPK/STAT3 pathway in human lung epithelial cells and mouse models infected with SARS-CoV (Li et al., 2016). Additionally, EGR1 is dysregulated in COVID-19 patient lung samples as identified *via* transcriptomic and bioinformatic analysis (Islam and Khan, 2020; Alexander et al., 2021; Xie et al., 2021).

Although, EGR1 has been shown to act in a proviral capacity for multiple coronaviruses (discussed above), there is also evidence that EGR1 has an antiviral role during HCoV-229E and PEDV infections. Yuan et al. found that EGR1 was upregulated following infection of H1299 cells with HCoV-229E. Knockdown of EGR1 in H1299 cells followed by infection with HCoV-229E resulted in significantly reduced VP-1 protein, viral mRNA, and PARP cleavage (Yuan et al., 2022). Additionally, in the section above the pro-viral role for EGR1 in PEDV infections was described. However, there appears to be conflicting evidence as to the role of EGR1 in these infections, some of these differences are likely due to the cells and conditions under which the role of EGR1 was examined. Wang et al. recently described an antiviral role for EGR1 following PEDV infection of LLC-PK1 cells, which are normal pig kidney cells and represent a more physiologically relevant model for studying PEDV. EGR1 was found to be upregulated at both the mRNA and protein level following infection with PEDV in LLC-PK1 cells (Wang et al., 2021). Furthermore, overexpression of EGR1 significantly decreased PEDV N protein as well as viral mRNA in Vero cells and these results were further confirmed using siRNA targeting EGR1 in which similar results were achieved (Wang et al., 2021). Additionally, mechanistic experiments involving ChIP assays revealed that EGR1 inhibits PEDV replication through direct binding of EGR1 to the IFN-regulated antiviral (IRAV) promoter to induce IRAV expression (Wang et al., 2021). Moreover, siRNA mediated knockdown of IRAV increased viral replication and microscopy studies revealed that IRAV colocalizes with PEDV N protein to facilitate its subsequent degradation and IRAV was found to be essential for EGR1 suppression of PEDV replication (Wang et al., 2021). Altogether, these data provide a more robust argument for EGR1 functioning in an antiviral capacity during PEDV infections.

#### Togaviridae

Viral and host transcriptomic studies identified EGR1 as being highly upregulated after infection with encephalitic Venezuelan equine encephalitis virus (VEEV) in U87MG cells, a human astrocytoma cell line (Baer et al., 2016). Next generation RNA sequencing (RNA-Seq) data was leveraged to elucidate clinically relevant alterations in the mRNA transcriptome of VEEV infected astrocytes. EGR1 was found to be differentially expressed after VEEV infection and results suggested that EGR1 may serve as a potential link between the innate immune response and unfolded protein response (UPR) pathways, two pathways intimately associated with apoptosis. Interestingly, loss of EGR1 inhibited VEEV-induced apoptosis but did not alter viral replication kinetics. Neuronal cell death is a hallmark of VEEV infection and VEEV induced neurological sequalae is common in survivors (Ronca et al., 2016). Additional studies indicated that EGR1 expression is upregulated in primary human astrocytes during VEEV infection and that EGR1 induction is dependent on ERK1/2 and protein kinase R (PKR)-like endoplasmic reticulum kinase (PERK; Dahal et al., 2020). More recent studies identified EGR1 dependent genes associated with inflammation, apoptosis, and/or encephalitis in VEEV infected astrocytes including ATF3, FOS, JUN, KLF4, EGR2, and EGR4 transcription factors and CXCL3, CXCL8, CXCL10, TNF, and prostaglandin synthase 2 (PTGS2) inflammatory mediators. Targeting of PTGS2 with the small molecule inhibitor celecoxib resulted in increased cell viability as compared to vehicle-treated infected cells (Lehman et al., 2022). Interestingly, EGR1 gene expression is also induced in U87MG cells infected with other alphaviruses, including eastern equine encephalitis virus (EEEV), chikungunya virus (CHIKV), and Sindbis virus (SINV; Lehman et al., 2022). EEEV infected cells displayed EGR1 dependent gene expression changes of ATF3, JUN, CXCL3, CXCL8, CXCL10, TNF, and PTGS2, similar to the results observed with VEEV. Additional studies are needed to further elucidate the underlying molecular mechanisms as to how EGR1 contributes to neuronal cell death following infection with VEEV.

#### Flaviviridae

EGR1 was identified as an activated host gene in *ex vivo* mouse brain samples from mice that were infected with Japanese encephalitis virus (JEV; Saha and Rangarajan, 2003). Following infection of astrocytes with Zika virus (ZIKV), a flavivirus associated with microcephaly and Guillain–Barré syndrome (Musso et al., 2019), EGR1 mRNA is upregulated approximately 2-fold (Lehman et al., 2022). Loss of function studies indicated that EGR1 at least partially regulates the expression of transcription factor KLF4 and inflammatory mediator TNF-α following ZIKV infection of astrocytes. However, to date, the full functional significance of EGR1 in JEV or ZIKV infections has yet to be determined.

#### Phenuiviridae

Rift Valley fever virus (RVFV) is a hemorrhagic fever virus known to induce significant hepatitis and encephalitis (Gaudreault et al., 2019). Infection of astrocytes with RVFV induced a robust induction of EGR1 mRNA (~70-fold over mock; Lehman et al., 2022). Further, loss of function studies indicated that EGR1 at least partially regulates the transcription of KLF4 and CXCL10 (Lehman et al., 2022). Given the sex differences in EGR1 expression and the abortion storms as a result of RVFV infection, it would be interesting to investigate if EGR1 plays any role in the manifestation of that disease phenotype.

## Discussion

As reviewed above, induction of EGR1 gene expression is a common theme amongst a wide variety of viruses, spanning from large DNA viruses to small RNA viruses. This is likely due to dsRNA and multiple cytokines, including IFNα, INFβ, IFNλ, and TNFα, being capable of increasing EGR1 transcription (Ramana and Das, 2020). However, there is a dearth of information on the impact of EGR1 on viral pathogenesis *in vivo*. This is somewhat surprising given the availability of EGR1 knockout mice (Lee et al., 1995), which have been used to elucidate the importance of EGR1 for other infectious diseases, including bacterial infections (Pang et al., 2019). Recent studies using Assay for Transposase-Accessible Chromatin (ATAC) sequencing have characterized EGR1 as a transcriptional regulator of inflammatory genes, suppressing the inflammatory response of macrophages (Trizzino et al., 2021). Complete loss of EGR1 using knockout mouse models has its limitations, and conditional knockout mice and/or therapeutics targeting EGR1 should be used as tools to more fully understand the impact of EGR1 on viral pathogenesis. To date there are no FDA approved small molecule inhibitors of EGR1 and only one research grade small molecule inhibitor of EGR1, AB1711, has been developed which blocks the ability of EGR1 to bind DNA (Yeo et al., 2021). Treatment of mice with AB1711 was able to prevent skin inflammation in a mouse model of atopic dermatitis. Future work should focus on elucidating the impact of EGR1 on viral pathogenesis especially as it relates to immune modulation and inflammatory processes.

Given the role of EGR1 in learning and memory (Duclot and Kabbaj, 2017; Gallo et al., 2018), the potential impacts of EGR1 on the development of neurological sequelae following viral infections is especially of interest. Particularly, the capability of EGR1 binding to methylated DNA and then mediating TET1 recruitment to demethylate target sequences may result in long-term epigenetic changes and altered cellular functions (Sun et al., 2019). Since epigenetic changes are reversible, it would be extremely valuable to the scientific community to precisely define the epigenetic aberrations associated with viral infections and further explore them as potential therapeutic targets. However, at the cellular level, biological systems are heterogeneous and viral infections may induce cell-type specific responses in individual cells. Growing evidence indicates that EGR1 may have distinct sets of target genes across tissues and thus play diverse roles in different types of cells. Although we have confirmed that EGR1 is able to bind to methylated DNA, it remains unclear the prerequisite(s) for EGR1 to achieve cell-type specific bindings. Mechanistic studies will aid in determining the causal links between EGR1 binding, histone modifications, and chromatin accessibility. Over the past decade, rapid development of single cell techniques have enabled the generation of multi-omics data simultaneously. In addition, recent advances in spatial sequencing technology enables obtainment of a high-resolution view of multicellular responses to viral infections in a three-dimensional manner without the loss of spatial information. Future single-cell plus spatial sequencing technologies will provide a better understanding of EGR1 functions within the highly complex tissue of the brain in response to viral infections and unlock novel EGR1-dependent epigenetic markers as therapeutic targets for drug development.

## Author contributions

CW and KK-H: conceptualization and writing—review and editing. CW: writing—original draft preparation. KK-H: funding acquisition. All authors contributed to the article and approved the submitted version.

## Funding

This work was funded through the Defense Threat Reduction Agency (DTRA) grant HDTRA1-21-1-0008 to KK-H: Funders do not have any role in the design of the study and collection, analysis, and interpretation of data and nor in writing the manuscript.

## Conflict of interest

The authors declare that the research was conducted in the absence of any commercial or financial relationships that could be construed as a potential conflict of interest.

## Publisher’s note

All claims expressed in this article are solely those of the authors and do not necessarily represent those of their affiliated organizations, or those of the publisher, the editors and the reviewers. Any product that may be evaluated in this article, or claim that may be made by its manufacturer, is not guaranteed or endorsed by the publisher.
